# The use of communication applications in orthopaedics: a scoping review

**DOI:** 10.1007/s00402-026-06318-z

**Published:** 2026-04-23

**Authors:** Annali Olivelle, Poojit Borra, Rami Sati, Mayek S Gupta, Krishna Oochit, Akash Patel

**Affiliations:** 1 University College London, London, UK; 2 Royal Free London NHS Foundation Trust, London, UK

**Keywords:** Communication applications, Orthopaedics, Mobile health, Clinician-to-clinician communication, Messaging applications

## Abstract

**Background:**

Effective communication between clinicians is vital in orthopaedic practice, particularly in trauma and emergency care where optimal decision making is necessary. Although communication application use in orthopaedics is common within practice, there is little research to back up its ongoing use. This scoping review aims to synthesise the existing evidence on the use of clinician-to-clinician communication applications in orthopaedic practice, focusing on its prevalence, perceived utility, diagnostic reliability and associated challenges.

**Materials and methods:**

A scoping review was conducted in accordance with the PRISMA-ScR guidelines. This involved a literature search of MEDLINE, Embase, and the Cochrane Library from inception to September 2025. Studies involving clinician-to-clinician communication applications used within orthopaedic care were included. Data was extracted and synthesised using a methodological approach.

**Results:**

Seven studies published between 2016 and 2023 were included. The evidence demonstrates widespread adoption of communication applications, particularly WhatsApp, for informal clinical communication, with consistent perceptions that these platforms improve communication efficiency and coordination of care. Four studies evaluating smartphone transmitted radiographic images reported good to excellent intraobserver diagnostic agreement when compared with Picture Archiving and Communication Systems (PACS). Significant limitations were also identified, including reduced image quality, physical usability constraints and inconsistent documentation resulting in substantial patient data governance and confidentiality concerns.

**Conclusion:**

Communication applications function as informal adjuncts to clinical communication in orthopaedic practice, driven by clinical necessity. While they support rapid consultation and decision making, their use remains limited by governance, data security, and integration challenges. Future efforts should focus on developing clinician centred communication systems that align usability with regulatory compliance, enabling safe and structured integration into orthopaedic care pathways.

## Introduction

 Effective communication between clinicians is fundamental to the safe and efficient delivery of orthopaedic care. It underpins every aspect of clinical practice, from the initial assessment and diagnostic imaging to surgical planning and post-operative management [1]. Within orthopaedic departments, multidisciplinary teams rely on seamless communication to coordinate complex patient pathways and ensure continuity of care across inpatient and outpatient settings. Traditionally, this has taken place through in person discussions, telephone calls or pagers however, as healthcare continues to evolve within an increasingly digital landscape, modern communication applications can add value through enhancing accessibility and improving clinical workflows [2].

Traditional communication methods often create substantial barriers to effective information exchange. They tend to be fragmented, lack integration across multidisciplinary teams, and rely heavily on synchronous communication, all of which can contribute to increased error rates and delays in patient care [3, 4]. These limitations have highlighted the need for more flexible and accessible solutions. Communication applications have been shown to improve communication between clinicians, facilitating faster and more efficient exchanges of information [5]. Surveys of medical staff and students indicate that a large majority regularly use messaging apps, email, or calls to discuss patient care, often finding text-based communication more efficient than phone calls or face to face meetings [6]. This highlights the need to incorporate digital communication within clinical practice, especially within the orthopaedic speciality.

Despite the widespread adoption of communication applications in healthcare, there remains limited research examining the use of these applications specifically within orthopaedic practice, including how clinicians employ these tools and perceive their role in facilitating communication and patient care [7]. Orthopaedic services typically involve large, multidisciplinary teams where effective and timely communication is crucial for ensuring patient safety and optimising outcomes [8]. This may involve anything from large virtual MDT calls to resident doctors escalating cases to offsite consultants. In any scenario, the requirements for orthopaedic consults tend to be more reliant on imaging and are also time critical, a requirement often fulfilled by communication applications.

However, the extent to which these applications are utilised in this context, as well as their perceived benefits and associated challenges, have not been comprehensively explored. While previous studies in broader clinical settings have demonstrated that forms of communication applications such as messaging applications can enhance communication efficiency and coordination, there is a lack of evidence focusing on orthopaedics specifically, despite the higher demand in this field [9]. The majority of reviews in the literature also focus on the patient to clinician aspect of communication applications, rather than the usage of these applications within a clinical team itself. Understanding how such applications are integrated into orthopaedic workflows and how clinicians perceive their utility is essential to guide future practice and policy within an increasingly digital healthcare system.

This scoping review aims to synthesise the existing evidence on the utility of communication applications within orthopaedic practice. Specifically, it will explore how and whether communication applications are being utilised by clinicians, the perceived benefits they offer in supporting decision making, coordination of care, diagnostic accuracy and efficiency of clinical management, as well as the barriers and challenges associated with their use. It will also consider clinicians attitudes and preferences towards communication applications, identifying whether these technologies are viewed as effective and sustainable tools for enhancing clinical practice.

## Methods

We analysed and summarised the existing data involving communication applications used by healthcare professionals in orthopaedics by conducting a scoping review of the literature. This review followed the methodological framework for scoping reviews outlined by Arksey and O’Malley, further refined by Levac et al. and was reported in accordance with the PRISMA-ScR (Preferred Reporting Items for Systematic Reviews and Meta-Analyses extension for Scoping Reviews) checklist [10, 11].

### Eligibility criteria

Inclusion criteria:

The inclusion criteria was defined according to the population concept context (PCC) framework recommended by the Joanna Briggs institute. Pollock et al. described the PCC framework as a ‘guide recommended to construct clear and meaningful objectives and eligibility criteria for a scoping review’ [12].

- Population: Clinicians involved in musculoskeletal (MSK) or orthopaedic care, including orthopaedic surgeons, physiotherapists, general practitioners managing MSK conditions and other healthcare professionals.
- Concept: Digital communication applications used to support clinical practice, consultation or collaboration in orthopaedics. This included mobile applications, web-based platforms and software designed for clinician-to-clinician communication.
- Context: Any clinical or healthcare setting where orthopaedic care is provided.

Studies were included if they reported on usability, acceptability, adoption, workflow impact, decision making or clinical utility of communication applications. No restrictions were placed on study design, publication date or geographical location. Only studies published in English were included.

Exclusion criteria:

Studies focussing exclusively on patient facing apps, non-digital communication interventions or those unrelated to orthopaedics or MSK practice.

### Search strategy and screening

The search strategy was developed in collaboration with a medical librarian to ensure a comprehensive and systematic approach to identifying relevant literature. The search was applied to the following electronic databases: MEDLINE (using OVID, 1946–present), Embase (using OVID, 1974–present), and the Cochrane Library (2005–present).

The strategy combined controlled vocabulary (e.g., MeSH terms) and free-text keywords related to *orthopaedics*, *communication applications*, and *clinicians*. Boolean operators and truncations were used to capture variations in terminology and spelling. The full search strategy for each database is provided in Appendix 1.

All databases were searched on 17/09/2025. Reference lists of included studies and relevant reviews were also screened manually to identify additional sources not captured in the electronic search.

**Fig. 1:**
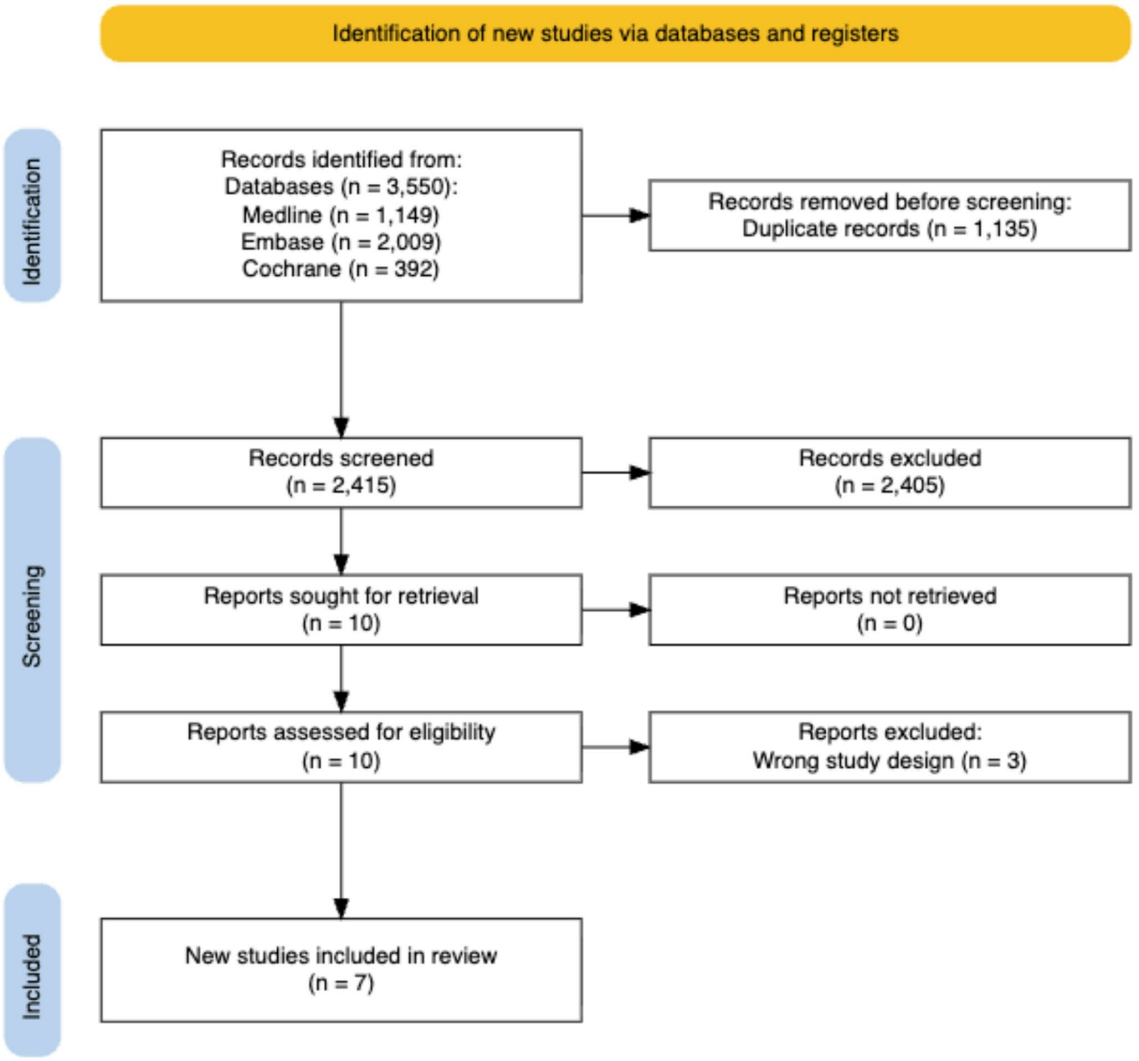
PRISMA-ScR flow diagram of the study selection process

All search results were imported into Rayyan a systematic review software, and duplicates were removed [13]. Two reviewers (AO, PB) independently screened titles and abstracts against the inclusion criteria. Full texts of potentially relevant studies were then reviewed for eligibility. Discrepancies after the initial abstract and title screening and full text screening were resolved through a third reviewer (MG). The selection process was documented using the PRISMA-ScR flow diagram (Fig. 1).

### Methodological analysis of included studies

In keeping with scoping review methodology, a formal risk of bias assessment was not performed. However, to support interpretation of the evidence, methodological limitations of included studies were descriptively summarised in Table 3.

## Data extraction

A structured data extraction table was created using Microsoft Excel 2019 (Microsoft Corporation, Redmond, WA) to systematically record key information from each included study. Two reviewers (RS) and (AO) independently extracted data from the final set of included studies, and two additional reviewers (MG, PB) verified the information to ensure accuracy and consistency.

No critical appraisal of study quality or risk of bias was performed. The included articles were read in full by two reviewers, who verified the extracted data and contributed to the synthesis process. A directed qualitative content analysis approach was used to organise and interpret the findings. Data were collated, summarised, and presented both narratively and in tabular format using Microsoft Word 2019 (Microsoft Corporation, Redmond, WA). Themes were identified around usability, acceptability, workflow integration, adoption, and clinical utility. The reviewers reached consensus on all extracted data and thematic classifications before final synthesis.

**Table 1:** Study characteristics

Author (year)	Country	Study design	Setting	Participants
Choemprayong et al. (2021) [14]	Thailand	Usability study	Medical school- simulation	5 orthopaedic physicians
McDonald et al. (2022) [15]	New Zealand	Audit + survey	Hospital	20 clinicians (9 house officers, 5 registrars, 2 fellows, 4 clinical nurse specialists)
Novoa-Parra et al. (2020) [16]	Spain	Pilot survey	Conference	41 orthopaedic surgeons (from 108 invited)
Stahl et al. (2016) [17]	Israel	Reliability study	Hospital	30 adults (≥ 18 years) with thoracolumbar spine fractures, evaluated by 5 senior orthopaedic spine surgeons
Paryavi et al. (2016) [18]	USA	Reliability study	Hospital	50 paediatric elbow cases assessed by 2 fellowship trained orthopaedic surgeons, 2 senior orthopaedic residents
Kapıcıoğlu et al. (2019) [19]	Turkey	Reliability study	Hospital	3 surgeons; 90 cases
Stahl et al. (2019) [20]	Israel	Reliability study	Hospital	5 surgeons; 73 cases

**Table 2:** Aims of included studies

Author (year)	Platform studied	Aims
Choemprayong et al. (2021) [14]	The smartphone app named “MEDIC” (a physician-to-physician teleconsultation system) on the iOS platform (iPhone) only	To assess the usability of the MEDIC teleconsultation app in orthopaedic practice using the PACMAD framework and identify areas for design improvement.
McDonald et al. (2022) [15]	WhatsApp	Assess prevalence of WhatsApp messaging, clinician attitudes, and awareness of mobile data protection policies
Novoa-Parra et al. (2020) [16]	WhatsApp	To evaluate the use of WhatsApp as a clinical tool among orthopaedic surgeons and to determine their perception of its usefulness in decision making
Stahl et al. (2016) [17]	WhatsApp instant messaging for smartphone video clips taken on iPhone 6 Picture Archiving and Communication System (PACS)	To compare intraobserver reliability of diagnosing, classifying (AO and Denis), and proposing treatment for thoracolumbar spine fractures between smartphone video clips sent via WhatsApp and standard PACS viewing
Paryavi et al. (2016) [18]	Smartphones (iPhone 5) capturing pictured radiographs, transmitted via MMS PACS	To assess the reliability of diagnosing paediatric elbow injuries using smartphone captured images versus PACS and to evaluate whether treatment decisions (operative vs. non-operative) differ between smartphone and PACS reviews.
Kapıcıoğlu et al. (2019) [19]	WhatsApp (iPhone 7 S photos of PACS images) PACS	To assess reliability of diagnosing paediatric supracondylar fractures using WhatsApp images vs. PACS and to evaluate whether diagnostic agreement with gold standard differs across WhatsApp and PACS
Stahl et al. (2019) [20]	WhatsApp (iPhone 5/6 photos of PACS images) PACS	To determine whether diagnosis, classification, and treatment decisions based on smartphone-captured radiographs are reliable compared with PACS

**Table 3:** Methodological Limitations of included studies

Author (year)	Methodological limitations
Choemprayong et al. (2021) [14]	Small sample size; Potential selection bias- all participants were iPhone users; Simulated environment; Lack of comparator group- the study did not compare MEDIC to existing communication tools. Observer and Hawthorne effect – participants were aware they were being observed and recorded, which may have influenced behaviour.
McDonald et al. (2022) [15]	Self-reported survey data; Potential observer bias in message classification – categorisation of messages was performed by researchers and may involve subjective interpretation; Limited depth of analysis – the study counted identifiable data and message types but did not explore contextual factors influencing inappropriate data sharing.
Novoa-Parra et al. (2020) [16]	Small effective sample size – although 108 participants were invited, the required sample size calculation indicated only 36 completed surveys were needed, limiting statistical power; Self-reported data – reliance on participants’ self-reported behaviours and perceptions introduces recall bias and social desirability bias. Questionnaire not formally validated – the survey was only piloted with two clinicians, with no formal psychometric validation reported. Potential non-response bias – characteristics of non-respondents were not analysed, which may influence results.
Stahl et al. (2016) [17]	Limited number of evaluators – only five surgeons participated, reducing external validity and increasing susceptibility to individual reviewer bias; Intraobserver design only – the study assessed agreement of each surgeon with themselves across two methods, but did not evaluate interobserver reliability between different clinicians; Recall bias- a four week interval between smartphone and PACS assessments may not fully eliminate recall bias, particularly for distinctive cases.; No assessment of clinical outcomes – the study measured agreement but did not evaluate whether smartphone- based decisions affected actual patient care
Paryavi et al. (2016) [18]	Limited number of evaluators – only four raters participated (two consultants and two residents), which may not represent the broader range of clinical experience. Recall bias – only a 1-week interval between PACS and smartphone assessments increases the risk of recall bias; Intraobserver reliability focus – the study primarily assessed agreement within the same raters rather than interobserver agreement across clinicians in real-world settings.
Kapıcıoğlu et al. (2019) [19]	Restricted patient population – only Gartland type 1 and 2 fractures and soft tissue injuries were included and more complex or severe fractures were excluded, limiting generalisability; Limited number of evaluators – only three orthopedic surgeons performed the WhatsApp assessments;
Stahl et al. (2019) [20]	Recall bias – 4-week interval between PACS and WhatsApp review may still allow memory bias; Limited number of evaluators – only 5 senior orthopedic surgeons participated, which may not capture broader clinical variability; Smartphone device variability – evaluators used different iPhone models (5/6), which could influence image quality and interpretation.

**Table 4:** Outcomes and utility perception of included studies

Author (year)	Measures assessed	Key findings	Perceived utility	Advantages	Limitations
Choemprayong et al. (2021) [14]	Effectiveness, efficiency, satisfaction, learnability, memorability, error, cognitive load	Tasks: (1) Group creation, (2) Patient registration, (3) Clinical encounter with simulated patient, (4) Form creation (clinical score), (5) Teleconsultation. Task completion: 100% for tasks 2–4, 80% for tasks 1 & 5; participants navigated more screens than expert; perceived difficulty 3–6/7; common errors in data input, navigation, and radiograph interpretation	Supports physician-to-physician teleconsultation; potential to improve workflow and professional collaboration	Novice users confident they could learn quickly potential to improve professional communication and collaboration task-based training useful	Small screen size/resolution navigation/icon misinterpretation typing/data errors difficulty interpreting radiographs cognitive load some tasks not completed
McDonald et al. (2022) [15]	Usage prevalence, message content, clinician attitudes, awareness of data security policies	1,360 messages sent over 2 weeks 28% contained patient-identifiable data clinicians rated use 9.1/10 65% unaware of data security policies	Supports efficient, non-hierarchical team communication facilitates rapid information sharing and coordination	Highly valued by clinicians enhances team coordination widely adopted supports rapid and effective communication	Data security risk (28% messages with patient data) 65% unaware of policies consumer app ownership raises issues (Meta/Facebook) bypasses formal record-keeping and audit trails need for governance and guidelines
Novoa-Parra (2020) [16]	Demographics, utility in clinical practice, perceived usefulness for clinical decisions	100% sent/received clinical info via WhatsApp 93% used it to consult cases 78% in WhatsApp groups 44% selected it as “clinical tool” indirectly	71% agreed/somewhat agreed it is useful for clinical decision-making WhatsApp perceived as established in orthopedic clinical practice	Rapid communication Image sharing Facilitates teamwork End-to-end encryption ensures message security	Small, regional sample Survey-based, self-reported Privacy/legal concerns (patient data) Limited generalisability
Stahl et al. (2016) [17]	Intraobserver agreement in diagnosing, classifying and recommending treatment using smartphone-transmitted images versus standard PACS viewing.	Fracture level: κ = 0.94 (near-perfect) AO classification: κ = 0.75 Denis classification: κ = 0.69 Treatment recommendation: κ = 0.73 Neural canal penetration: κ = 0.71 Loss of vertebral height: κ = 0.55 Kyphosis: κ = 0.45 (moderate) No fractures missed using smartphone video	Smartphone videos provide reliable evaluation comparable to standard PACS for diagnostic and treatment decisions	Rapid, inexpensive, and widely accessible Enables offsite consultations Reduces unnecessary patient transfers Useful in rural or community emergency settings Simple to implement with existing smartphones and WhatsApp	Small, single-center study Potential recall bias despite 4-week interval Smartphone limitations: smaller screen, lower resolution, no PACS features (zoom, window-level adjustment) Not suitable for all treatment decisions- PACS recommended for surgical planning Only thoracolumbar fractures studied- results may not generalize to other spine pathologies
Paryavi (2016) [18]	Intraobserver reliability of the Diagnostic classification of paediatric elbow injuries (6 categories) and treatment recommendation (operative vs. non-operative)	Excellent intraobserver reliability for diagnosis: κ = 0.89–0.94, Overall κ = 0.91 for all raters Treatment decision κ = 0.80–0.916, Overall κ = 0.86	High – smartphone images provide reliable evaluation comparable to PACS for urgent clinical decision-making	Rapid, accessible, and inexpensive Enables offsite or emergent consultation Effective even for challenging/minimally displaced injuries Can streamline emergency care and reduce delays	Small, single-centre study Only 4 evaluators MMS image quality dependent on smartphone camera and monitor Not a replacement for in-person evaluation Limited generalizability to other injuries or anatomical locations Health Insurance Portability and Accountability Act (HIPAA)/privacy concerns if not de-identified
Kapıcıoğlu (2019) [19]	Diagnostic classification into: soft tissue injury, Gartland type I, Gartland type II Comparison across 3 rounds: WhatsApp (round 1), WhatsApp (round 2), PACS (round 3) Intraobserver reliability Agreement with gold-standard PACS diagnosis	Agreement between WhatsApp evaluations and PACS was good to very good (κ = 0.74–0.88). Intraobserver reliability ranged from moderate to very good (κ = 0.55–0.80). Agreement with gold-standard diagnosis was almost very good to very good (κ = 0.78–0.96).	High- Overall WhatsApp images provided highly reliable diagnosis of type I–II paediatric supracondylar fractures compared with PACS.	Fast, accessible, low-cost Supports urgent/emergency decision-making High diagnostic agreement even with reduced image resolution Potential to reduce ED wait times and unnecessary transfers	Recall bias due to repeated evaluations WhatsApp image compression reduces resolution by ~⅓ No image manipulation (contrast/zoom) compared to PACS Only lateral views assessed Not a substitute for in-person exam Small sample per injury type
Stahl (2019) [20]	Intraobserver agreement (Cohen’s κ) for diagnosis, fracture classification, and management decisions and agreement across anatomical regions	Near-perfect overall agreement between smartphone and PACS: Diagnosis: κ = 0.87 Classification: κ = 0.83 Treatment choice: κ = 0.87	Highly reliable tool for remote orthopedic consultation in pediatric trauma; useful when specialists are off-site	Fast, accessible remote decision-making High diagnostic reliability comparable to PACS. Rapid image sharing facilitating timely consultation. Useful for ED triage and reducing unnecessary transfers. Cost-effective (no special apps needed).	Lower image quality vs. PACS (screen reflection, focus issues, lower resolution). Cannot manipulate images (contrast, brightness, fine zoom). Recall bias possible. Simulated rather than real ED conditions.

## Results

### Selection of sources of evidence

The final literature search yielded 3,550 studies till September 2025, of which 1,135 were duplicates. Once the remaining 2,415 underwent Title and Abstract screening as well as a Full Text screening, seven articles remained. The studies that were excluded at the full text screening stage, were done so due to their study design either being too broad or focusing on a different aspect of communication applications.

### Characteristics of included studies

The included studies were published between 2016 and 2023, conducted in Thailand, New Zealand, Spain, Israel, USA and Turkey [14–20]. Four of these studies followed a reliability study structure, two were cross-sectional surveys, and one was a usability study. All studies were primarily composed of orthopaedic trainee surgeons. A summary of the study characteristics can be seen in Table 1.

### Overview of results

The included studies were grouped into three categories based on their methodology. A methodological approach was chosen over thematic analysis due to the heterogeneity of study designs across the included literature. Where similar findings were identified across different study types, these were reported within their respective methodological categories in order to preserve the context of the original study design.

The first category comprised reliability studies (Kapicioglu et al., Stahl et al., Stahl et al., and Paryavi et al.), which evaluated diagnostic and decision-making agreement between messaging platforms and PACS [17–20]. The second category included cross-sectional surveys (Novoa-Parra et al. and McDonald et al.), which explored clinician perceptions and patterns of use [15, 16]. The final category consisted of usability studies, represented by Choemprayong et al., which examined the practical application and user experience of communication tools in clinical settings [14].

### Reliability studies

#### Diagnostic reliability

**Fig. 2:**
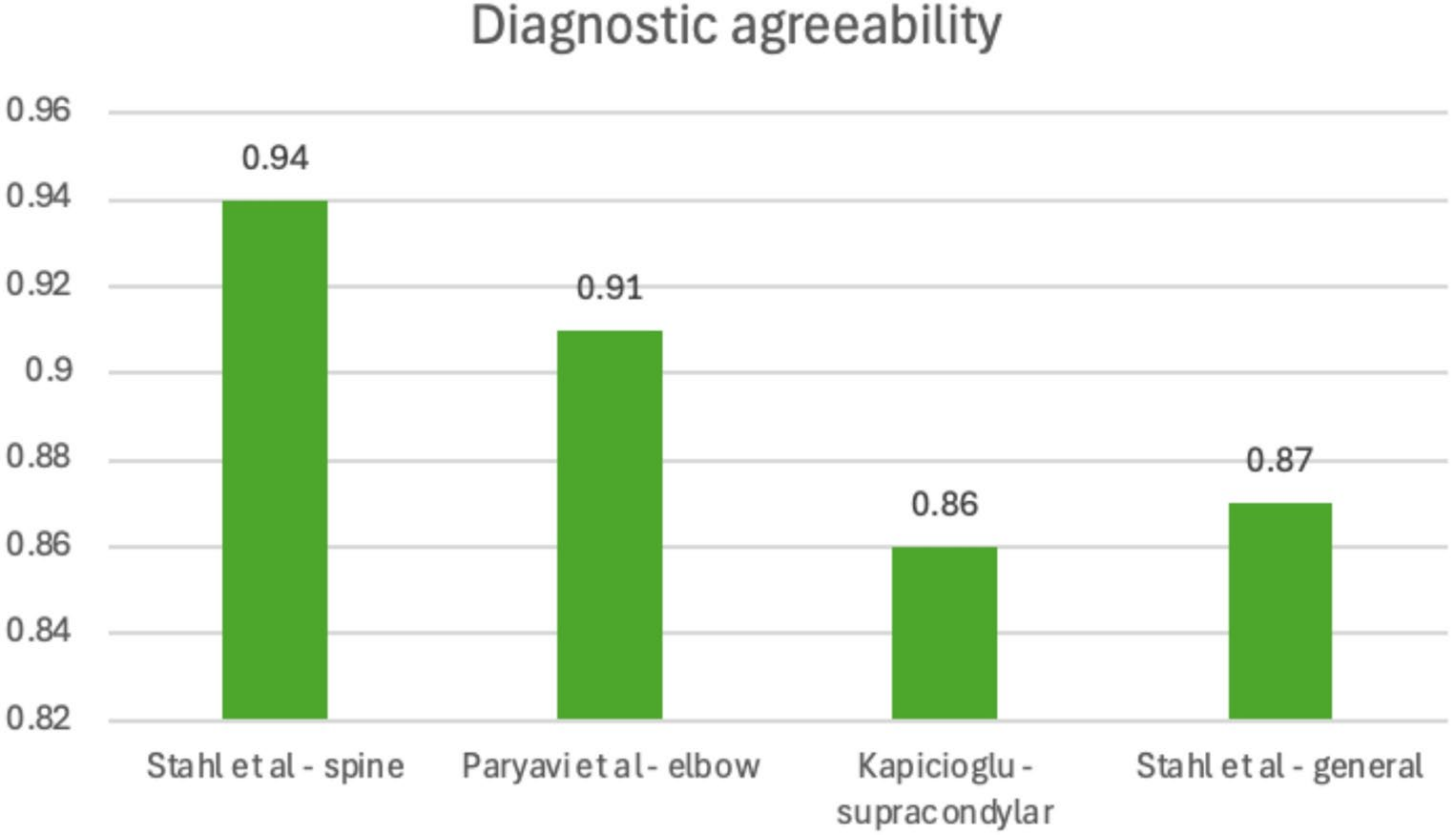
Diagnostic agreement between WhatsApp-shared images and PACS

Four of the included studies focused on comparing the reliability of diagnosing pathology from images sent over WhatsApp to looking at PACS directly [17–20]. Although each study focused on different types of fractures, all studies found a high kappa (k) intraobserver diagnostic agreement: (Stahl et al. k = 0.94 (0.89–0.98); Paryavi et al. k = 0.91 (0.855-0-0.954); Kapicioglu et al. k = 0.86 (no CI reported); Stahl et al. k = 0.87 (0.83–0.91)). Kappa values were above 0.8 in all studies, suggesting excellent diagnostic agreement between using WhatsApp and PACS [21]. These results are shown in Fig. 2.

**Fig. 3:**
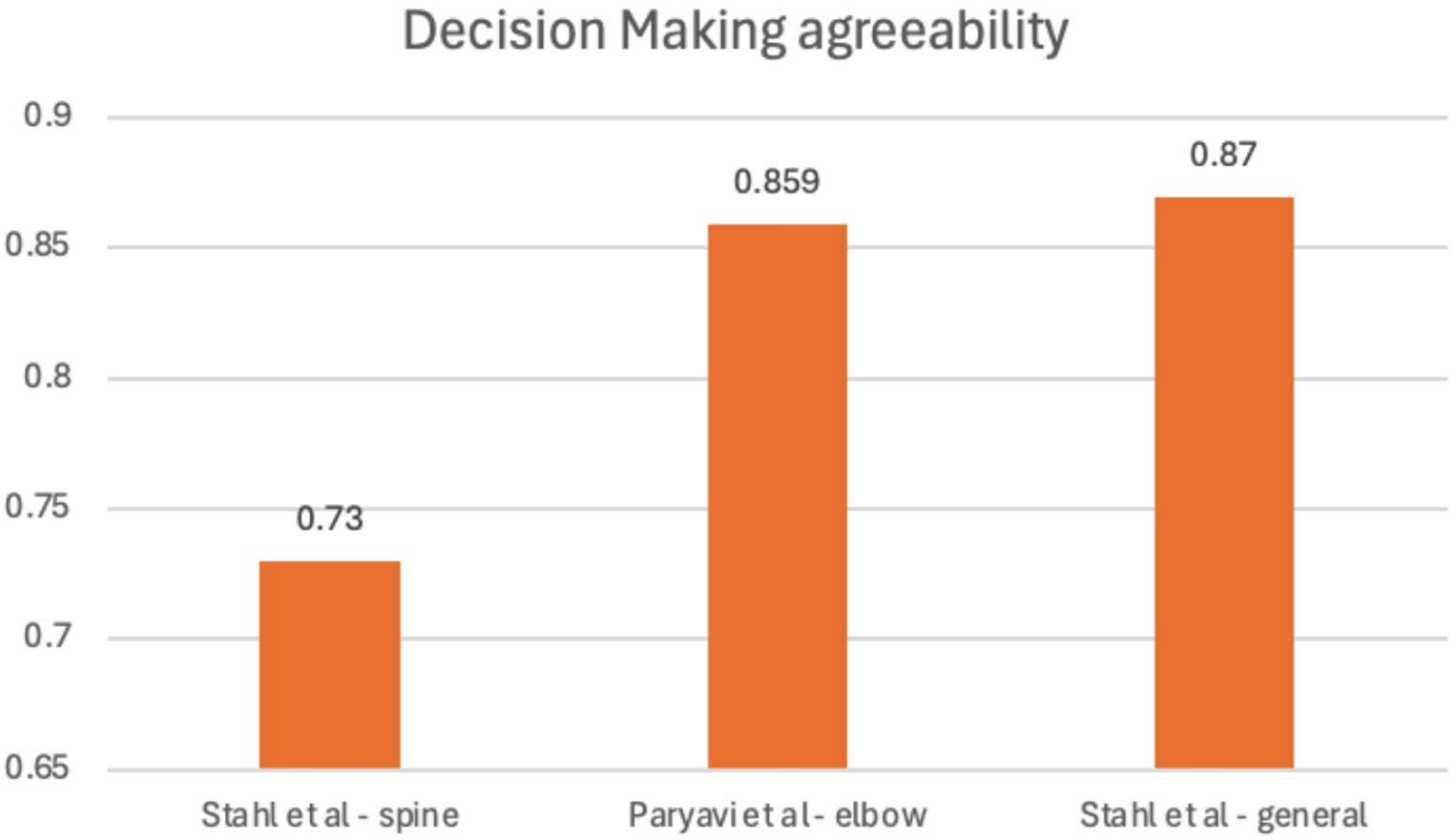
Agreement in treatment decision making based on WhatsApp shared images compared with PACS

Three out of the four studies comparing the diagnostic accuracy of PACS to WhatsApp also reported data on decision making accuracy in terms of management of the perceived diagnosis [17, 18, 20]. Although lower than the values for diagnostic agreeability, the kappa values for decision making also remained largely in the good to very good range (Stahl et al. k = 0.73 (0.62–0.84); Paryavi et al. k = 0.859 (0.786–0.997); Stahl et al. k = 0.87 (0.82–0.93)). These results are shown in Fig. 3.

#### Physical usability challenges

Kapicioglu et al. stated difficulties with the inability to see fine details on smaller screens and further mentioned that the quality and resolution of images on the smartphones were mostly inferior to that of the original X-ray [19]. This may have been due to the function of WhatsApp’s image sharing reducing the image resolution. Images sent over WhatsApp also lacked the feature of image manipulation such as changing contrast, magnification and rotation, which can often be done on PACS. These concerns were echoed by Stahl et al. and Paryavi et al., with Stahl et al. also additionally mentioning problems with image capture, resulting in excessive screen reflection and loss of focus [17, 18].

#### Patient data concerns

Paryavi et al. briefly mentioned that HIPAA compliance may be an issue but in this study all images in the study were de-identified and deleted. They further recommended that any image sharing must comply with HIPAA regulations but did not delve deeper into looking at the practicalities of doing so or the prevalence of data safety practices among clinicians [18].

### Cross-sectional surveys

#### Improved clinical communication

Messaging applications were found to facilitate rapid communication between specialities, mostly through an informal route. Novoa-Parra et al., found that in a survey of 41 orthopaedic surgeons, 71% of them agreed or somewhat agreed that ‘communication between clinicians through WhatsApp is a useful tool for making therapeutic decisions [16]. This was corroborated by McDonald et al., who found that the highest reported benefit of using WhatsApp was the convenience of being able to communicate with all team members in one forum, making it much easier to keep up to date with patient progress [16]. The participants in this study gave WhatsApp a usefulness rating of 9.1/10.

#### Patient data concerns

Concerns regarding patient data protection and confidentiality were previously briefly mentioned in the reliability studies. However, the paper that brought the biggest focus on data concerns was McDonald et al., who found that 28% of messages sent contained identifiable data, with 65% of clinicians surveyed unaware about data policies and 50% stating they allow other individuals to access their phone [15]. This meant that consent for photograph sharing was not clearly documented in clinical notes and photos were not deleted after use, alongside frequent access to these devices by individuals outside of the patient’s care.

A contributing factor to the concerns with patient data, is the lack of formal integration of these tools into the clinical technology ecosystem.

McDonald et al. found that this resulted in a lack of formal record keeping of any consent obtained in regard to pictures taken (only 45% of participants did), documentation of decisions made and any advice given (only 45% of participants did) [15]. All participants did not view WhatsApp as a formal medical record.

### Usability studies

#### Improved clinical communication

Choemprayong et al., developed their own messaging application (MEDIC) to help with group formation, patient registration, clinical data capturing, case record form creation and teleconsultation with other physicians [14]. After testing, all participants felt that the app would improve the efficiency of consultation. The app was preferred to instant messengers, such as Whatsapp, for clinical discussions as they offered better organisation.

#### Physical usability challenges

In the Choemprayong et al. study, there was significant reporting of the difficulties with using a smartphone for image interpretation. Clinicians in this study blamed the small size of the mobile phone, which resulted with 4 out of 5 missing a fracture on a hip x-ray, despite enlarging the image. Additional screen size and resolution limitations were also blamed for icon ambiguity, typing difficulties and a higher cognitive load while trying to perform a consultation [14].

### Summary of results

A complete summary of included studies by methodology and reported outcomes can be seen in Table 5.

**Table 5:** Summary of included studies by methodology and reported outcomes

Study design	Study included	O1	O2	O3	O4
Reliability Studies	Stahl et al. [17]	–	✓	✓	✓
	Paryavi et al. [18]				
	Kapıcıoğlu et al. [19]				
	Stahl et al. [20]				
Cross sectional surveys	Novoa-Parra et al. [16]	✓	–	–	✓
	McDonald et al. [15]				
Usability Studies	Choemprayong et al. [14]	✓	–	✓	–

Outcome domains: O1 = Improved clinical communication, O2 = Good remote diagnostic reliability, O3 = Physical usability challenges, O4 = Patient data concerns

## Discussion

### Overview

This scoping review highlights the increasing use of communication applications within orthopaedic practice, demonstrating both their perceived clinical utility and their potential limitations. Given the methodological heterogeneity of the included studies, findings were interpreted according to study design, distinguishing between evidence relating to diagnostic reliability, clinician perception and adoption, and usability. Reliability studies suggest that smartphone-based image sharing may provide good intraobserver agreement when compared with PACS, supporting its potential role in remote clinical decision making [17–20]. In contrast, survey based studies emphasise the widespread adoption and perceived convenience of these platforms, while usability data highlight practical challenges associated with smartphone interfaces [14–16]. Taken together, these findings suggest that communication applications fulfil an important role in facilitating rapid clinical interaction, but that current evidence primarily reflects diagnostic agreement and user perception rather than validated improvements in patient outcome.

Despite the search being conducted in September 2025, there is an absence of included studies published between 2023 until the date. Studies published during this period predominantly focused on emerging technologies such as artificial intelligence driven decision support systems and patient–clinician interfaces, which fell outside the scope of this review. Specifically, many were excluded based on the predefined PCC framework, as they centred on patient facing applications or automated clinical tools rather than clinician-to-clinician communication via communication applications. This suggests that while research in digital health continues to expand rapidly, there remains a relative lack of recent literature specifically addressing the use of communication applications for clinician use within orthopaedics.

### Clinical utility and reliability

The reliability studies in this review indicate that sharing images via smartphones can achieve a high degree of intraobserver diagnostic consistency compared with PACS, with consistently strong kappa values observed across various fracture types [17–20]. This means that individual clinicians are able to reproduce their own diagnostic decisions when interpreting images via communication applications, supporting their potential role in facilitating remote clinical input. Furthermore, agreement in treatment decision making, although slightly lower than diagnostic agreement, remained within the ‘good to very good’ range. This highlights that while communication applications may extend beyond diagnosis to support management planning, there is greater variability in how clinicians translate similar diagnostic interpretations into treatment decisions. This creates an additional layer of clinical complexity that is not fully captured by diagnostic reliability alone. However, these findings must be interpreted with caution. All included studies assessed only intraobserver reliability, with no evaluation of interobserver agreement. In clinical practice, where communication applications are frequently used to seek second opinions or support multidisciplinary decision making, consistency between different clinicians is arguably more important. This absence therefore limits the generalisability of these findings and weakens the extent to which a high diagnostic agreement can be applied in real world settings.

The survey based studies further support the use of communication applications, particularly in facilitating rapid and efficient communication between clinicians [15, 16]. High levels of perceived usefulness were reported, with clinicians highlighting the ability to share information within a single platform and remain updated on patient progress as key advantages [15]. This shows that beyond diagnostic agreement, communication applications play an important role in streamlining multidisciplinary communication and supporting timely clinical decision making. The development of purpose-built applications, such as MEDIC, further reinforces this need, with users favouring platforms that offer improved organisation and integration of clinical information over conventional messaging services [14]. This highlights that while existing applications are widely adopted, there remains a demand for more structured and clinically tailored communication systems.

The nearly unanimous adoption of WhatsApp and other communication applications despite the lack of formal appreciation suggests that they are meeting an unmet need. This need likely reflects the demand for rapid and flexible communication, particularly in emergency care settings [22]. This is further compounded by the corroborated findings across studies from 7 different countries, suggesting that there is a global system wide gap in formal communication infrastructure within orthopaedics.

An important consideration is that the majority of included studies focused exclusively on WhatsApp. WhatsApp’s frequent use may reflect familiarity and convenience rather than inherent superiority, which could bias perceptions of its utility and reliability [23, 24]. Furthermore, other messaging applications may differ in functionality or user interface which could affect both diagnostic reliability and workflow efficiency [25]. As such, the findings may not be fully generalisable to alternative platforms increasingly used in clinical practice.

### Technical and practical limitations

All the reliability studies comparing PACS to smartphone-based interpretation, found there to be physical and technical limitations to using shared images of scans rather than the imaging software itself [17–20]. For example, small screen sizes, image resolution and the lack of ability to manipulate images may limit the ability to detect pathology [19]. Multiple studies identified the inability to view images taken from PACS, in different viewpoints, potentially resulting in misdiagnosis. Although altogether unsurprising, it doesn’t limit its popularity among physicians. This may suggest that despite the image quality of the smartphone shared images being subpar, it is still an important tool to facilitate rapid discussion among physicians. It should be emphasised that WhatsApp is used as an adjunct with official referral pathways, rather than as a replacement [26, 27].

Usability data demonstrated that clinicians may fail to detect clinically significant pathology when using smartphone interfaces, with the majority of participants in one study missing a hip fracture despite image enlargement [14]. Such findings highlight the potential for missed diagnoses, particularly in cases requiring detailed image interrogation or involving subtle abnormalities.

### Medico-legal concerns and real world practice

A prominent challenge identified across studies relates to medico-legal and data governance concerns [15, 16, 28]. Using communication applications such as consumer messaging platforms for sharing clinical information raises issues regarding patient confidentiality, data ownership and regulatory compliance [29]. Furthermore, recent legislation, including the Online Safety Act 2023, gives Ofcom powers to monitor messages, which could weaken end-to-end encryption and compromise patient privacy during transit if the proper precautions to anonymise patient data is not taken [30]. These concerns are particularly relevant considering the General Medical Council (GMC) guidance. The GMC emphasises that professional standards, including the duty to maintain patient confidentiality, apply equally to digital and social media communication [31]. According to the Medical Defence Union (MDU), the inability to comply with these standards, whether inadvertently through communication applications, ‘can result in you facing a patient complaint, a disciplinary investigation by your employer or a GMC investigation’ [32]. Clinicians should therefore prioritise the use of secure, approved communication platforms, ensure patient information is anonymised wherever possible and adhere strictly to local and national data governance policies when using communication applications.

Clinicians have both an ethical and legal duty to protect patient information and must ensure that any disclosures are justified and compliant with confidentiality standards [33]. Clinicians can share patient information with others involved in a patient’s direct care when necessary to provide safe and effective treatment, but they remain responsible for safeguarding personal information [33–35]. The use of communication applications to share patient identifiable data introduces additional risks, as information can be forwarded to incorrect people, stored insecurely and simply misread. These risks are amplified in large professional group chats, where information may be rapidly disseminated beyond intended recipients, increasing the likelihood of breaches and potential exposure to individuals outside the clinical team. A notable example of this occurring was in the reprimand issued by the Information Commissioner’s Office (ICO) to NHS Lanarkshire, where 26 staff members shared patient data via a WhatsApp group between April 2020 and April 2022 [36, 37]. A non-staff member was also inadvertently added to the group, resulting in unauthorised disclosure. The ICO found that the organisation lacked appropriate policies, guidance, and risk assessment processes to manage communication application use. In this case, regulatory action was taken at an organisational level, with the ICO issuing a reprimand to the health board as the data controller, rather than to individual clinicians. However, staff involved were subject to an internal investigation, retraining, and governance measures [36]. In terms of legal accountability, under UK GDPR the primary responsibility for lawful data processing rests with the organisation as the data controller. However, this does not remove individual clinician responsibility, as healthcare professionals remain personally accountable under professional regulatory frameworks such as GMC guidance for maintaining patient confidentiality. Therefore, while enforcement action for data breaches is typically directed at organisational level, individuals involved may still be subject to internal disciplinary procedures, professional investigation, and fitness to practise scrutiny. The use of group messaging environments does not dilute this responsibility, and each user remains accountable for their own handling and disclosure of patient information.

Despite incidents like this, there is still no official NHS guidance on the use of certain communication applications in practice [32].

### Practice vs. policy

A finding of interest made by McDonald et al. [15], is that some clinicians used these communication applications despite an understanding of their legal ramifications. Many applications used do not comply with local data security regulations or international frameworks such as the GDPR, and their continued use puts patient data at risk [28]. Further to this point, many also show an understanding that these informal consultations are not part of the formal workflow. This results in a lack of documentation [15]. It is important to understand the reasons for why this paradox between understanding and action exists, with the literature indicating that the perceived usefulness simply outweighs any regulatory concerns [38]. It is potentially the case that due to this high reliability of informal consultations in more acute settings of patient care, any data concerns are not as heavily considered. The high perceived utility and reliability of these tools may further reinforce their use, particularly in acute care environments where timely decision-making is prioritised. As a result, non-compliant practices may become normalised within clinical teams.

Importantly, this disconnect suggests that the issue extends beyond individual clinician behaviour and may instead reflect limitations in existing institutional communication systems, which often fail to provide equally efficient and accessible alternatives.

### Integration and system challenges

Several studies reported the transmission of patient identifiable information without consistent documentation of consent [15, 16, 28]. The nature of these commercial tools does make messaging applications efficient and attractive for clinical communication however, they undermine formal documentation and accountability. Decisions made via these communication applications may not be appropriately documented, officially leading to unclear responsibility for clinical decisions [39] .

There is limited awareness among clinicians of institutional data protection polices therefore clinicians and healthcare organisations can be exposed to potential legal and ethical risks [15, 39]. However, our review suggests that these issues may reflect a system level failure rather than purely due to individual clinician behaviour. This interpretation is supported by the consistent finding across included studies that clinicians continue to use consumer messaging applications for clinical communication despite awareness of confidentiality risks and the absence of formal approval [15]. The use of these communication tools and consumer messaging systems are widely adopted because of the practicality and efficiency of communication that address real clinical needs [24]. The continued use of communication applications and perceived utility by clinicians, despite policy misalignment, indicates that clinical practice is evolving more rapidly than formal governance frameworks.

Although our review highlights the widespread adoption and perceived value of communication applications within orthopaedic settings, their use remains insufficiently validated to be formally utilised [23]. Communication applications such as commercial communication applications are not endorsed or regulated for clinical use [23]. This creates a disconnect between evidence of functional utility as seen from these studies, and institutional recognition, which limits their integration into standard care pathways. Several studies noted poor adoption of hospital approved messaging platforms that prioritise regulatory compliance over clinical usability [14, 15]. Although these systems are often designed to mitigate medico-legal risk, they may be inefficient and not aligned with clinical workflows often lacking group messaging functions and having inconvenient login steps. As a result, clinicians frequently revert to familiar consumer applications that offer a greater ease of use.

### Future considerations

Future work should focus on integrating clinicians feedback into useable communication tools that encompass the efficiency and usability of commercial messaging applications whilst integrating data protection policies. In parallel, institutional policies should be developed to reflect this real work usage and provide clear guidance on the acceptable use of commercial applications by possibly integrating documentation requirements and data protection responsibilities. There is also a personal responsibility for individual practitioners to practice secure data sharing, which can be enhanced through better education and training initiatives.

In addition, future research should move beyond reliability studies and surveys to evaluate clinical outcomes. While existing studies demonstrate high diagnostic agreement and perceived utility, they do not establish whether the use of these tools translates into improved patient outcomes or safer decision making. There should be more objective data to justify the use of communication applications for use in clinical settings, particularly within orthopaedics.

## Conclusion

This scoping review highlights that communication applications currently function as informal tools yet are perceived by clinicians as useful for facilitating rapid communication. Across healthcare settings, clinicians consistently use messaging platforms to triage, seek rapid advice and coordinate care, particularly in time critical and out-of-hours scenarios. It is known that clinicians rely on consumer communication applications which reflects a current misalignment between clinical workflows and institutional communication systems, with clinicians favouring usability over regulatory compliance. This raises ongoing concerns regarding patient confidentiality, data security, and compliance with institutional and national information governance policies when such platforms are used in clinical practice. However, none of the included studies evaluated their impact on patient outcomes. Therefore, while these tools may support workflow and decision making, claims regarding clinical benefit should be made cautiously, and further research is needed to establish their effect on patient care.

## Appendix 1

### Search terms by database

#### Cochrane

1. “Orthopedic Surgeons”:ti, ab, kw.
2. orthopaedic: ti, ab, kw OR orthopedic: ti, ab, kw OR musculoskeletal: ti, ab, kw.
3. clinician*:ti, ab, kw OR (health NEXT care NEXT professional*):ti, ab, kw OR (healthcare NEXT professional*):ti, ab, kw OR doctor*:ti, ab, kw OR physician*:ti, ab, kw OR surgeon*:ti, ab, kw OR (medical NEXT staff): ti, ab, kw.
4. (orthopaedic OR orthopedic OR musculoskeletal) NEAR/3 (decision* OR diagnos* OR treatment OR care OR plan*):ti, ab, kw.
5. #1 OR (#2 AND #3) OR #4.
6. “Mobile Applications”:ti, ab, kw.
7. “computer communication networks”:ti, ab, kw OR “internet use”:ti, ab, kw OR “internet-based intervention”:ti, ab, kw OR “web browser”:ti, ab, kw.
8. “user-computer interface”:ti, ab, kw.
9. ((mobile OR smartphone* OR web-based OR web OR online OR internet OR app* OR digital OR electronic OR e-health OR ehealth) NEAR/3 (tool* OR application* OR software OR platform* OR program* OR system* OR interface*)):ti, ab, kw.
10. #6 OR #7 OR #8 OR #9.
11. utility: ti, ab, kw OR usability: ti, ab, kw OR “user experience”:ti, ab, kw OR UX: ti, ab, kw OR “user satisfaction”:ti, ab, kw OR acceptability: ti, ab, kw OR adoption: ti, ab, kw OR feasibility: ti, ab, kw OR “clinical workflow”:ti, ab, kw OR workflow: ti, ab, kw OR “practice change”:ti, ab, kw OR “practice transformation”:ti, ab, kw OR “impact on care”:ti, ab, kw OR “impact on practice”:ti, ab, kw OR (shared NEXT decision*):ti, ab, kw OR (treatment NEXT outcome*):ti, ab, kw OR (professional-patient NEXT relation*):ti, ab, kw OR implementation: ti, ab, kw OR effectiveness: ti, ab, kw OR “quality improvement”:ti, ab, kw OR “process improvement”:ti, ab, kw OR “care improvement”:ti, ab, kw OR “service improvement”:ti, ab, kw OR (patient-centred NEXT care): ti, ab, kw OR (patient-centered NEXT care): ti, ab, kw OR (end-user NEXT experience): ti, ab, kw OR (stakeholder NEXT experience): ti, ab, kw OR (patient NEXT engagement): ti, ab, kw OR (professional NEXT acceptance): ti, ab, kw OR (provider NEXT adoption): ti, ab, kw OR (integration NEXT into NEXT practice): ti, ab, kw OR (real-world NEXT use): ti, ab, kw OR (routine NEXT practice): ti, ab, kw OR (clinical NEXT uptake): ti, ab, kw OR (system NEXT usability): ti, ab, kw.
12. (communication NEXT app*):ti, ab, kw OR (messaging NEXT app*):ti, ab, kw OR (text NEXT message*):ti, ab, kw OR SMS: ti, ab, kw OR (instant NEXT message*):ti, ab, kw OR (communication NEXT tool*):ti, ab, kw OR (communication NEXT platform*):ti, ab, kw OR (communication NEXT software): ti, ab, kw OR (online NEXT portal*):ti, ab, kw OR (web NEXT portal*):ti, ab, kw.
13. #10 OR #12.
14. #5 AND #11 AND #13.

#### Medline

1. Orthopaedic Surgeons/.
2. (orthopaedic or orthopedic or musculoskeletal).ti, ab, kw, kf.
3. (clinician* or “health care professional*” or “healthcare professional*” or doctor* or physician* or surgeon* or “medical staff”).ti, ab, kw, kf.
4. ((orthopaedic or orthopedic or musculoskeletal) adj3 (decision* or diagnos* or treatment or care or plan*)).ti, ab, kw, kf.
5. 1 or (2 and 3) or 4.
6. Mobile Applications/.
7. computer communication networks/ or “internet use”/ or internet-based intervention/ or web browser/.
8. exp user-computer interface/.
9. ((mobile or smartphone* or web-based or web or online or internet or app* or digital or electronic or e-health or ehealth) adj3 (tool* or application* or software or platform* or program* or system* or interface*)).ti, ab, kw, kf.
10. 6 or 7 or 8 or 9.
11. (utility or usability or “user experience” or UX or “user satisfaction” or acceptability or adoption or feasibility or “clinical workflow” or workflow or “practice change” or “practice transformation” or “impact on care” or “impact on practice” or “decision making” or “shared decision*” or “treatment outcome*” or “professional-patient relation*” or implementation or effectiveness or “quality improvement” or “process improvement” or “care improvement” or “service improvement” or “patient-centred care” or “patient-centered care” or “end-user experience” or “stakeholder experience” or “patient engagement” or “professional acceptance” or “provider adoption” or “integration into practice” or “real-world use” or “routine practice” or “clinical uptake” or “system usability”).ti, ab, kw, kf.
12. (“communication app*” or “messaging app*” or “text message*” or SMS or “instant message*” or “communication tool*” or “communication platform*” or “communication software” or “online portal*” or “web portal*”).ti, ab, kf.
13. 10 or 12.
14. 5 and 11 and 13.
15. limit 14 to English.

#### Embase

1. Orthopaedic Surgeons/.
2. (orthopaedic or orthopedic or musculoskeletal).ti, ab, kw, kf.
3. (clinician* or “health care professional*” or “healthcare professional*” or doctor* or physician* or surgeon* or “medical staff”).ti, ab, kw, kf.
4. ((orthopaedic or orthopedic or musculoskeletal) adj3 (decision* or diagnos* or treatment or care or plan*)).ti, ab, kw, kf.
5. 1 or (2 and 3) or 4.
6. Mobile Applications/.
7. computer communication networks/ or “internet use”/ or internet-based intervention/ or web browser/.
8. exp user-computer interface/.
9. ((mobile or smartphone* or web-based or web or online or internet or app* or digital or electronic or e-health or ehealth) adj3 (tool* or application* or software or platform* or program* or system* or interface*)).ti, ab, kw, kf.
10. 6 or 7 or 8 or 9.
11. (utility or usability or “user experience” or UX or “user satisfaction” or acceptability or adoption or feasibility or “clinical workflow” or workflow or “practice change” or “practice transformation” or “impact on care” or “impact on practice” or “decision making” or “shared decision*” or “treatment outcome*” or “professional-patient relation*” or implementation or effectiveness or “quality improvement” or “process improvement” or “care improvement” or “service improvement” or “patient-centred care” or “patient-centered care” or “end-user experience” or “stakeholder experience” or “patient engagement” or “professional acceptance” or “provider adoption” or “integration into practice” or “real-world use” or “routine practice” or “clinical uptake” or “system usability”).ti, ab, kw, kf.
12. (“communication app*” or “messaging app*” or “text message*” or SMS or “instant message*” or “communication tool*” or “communication platform*” or “communication software” or “online portal*” or “web portal*”).ti, ab, kf.
13. 10 or 12.
14. 5 and 11 and 13.
15. limit 14 to English.

## Data Availability

No new datasets were generated or analyzed for this study. All data used are publicly available from the cited sources in the manuscript.
